# Trends in paediatric and adult bloodstream infections at a Ghanaian referral hospital: a retrospective study

**DOI:** 10.1186/s12941-016-0163-z

**Published:** 2016-08-18

**Authors:** Noah Obeng-Nkrumah, Appiah-Korang Labi, Naa Okaikor Addison, Juliana Ewuramma Mbiriba Labi, Georgina Awuah-Mensah

**Affiliations:** 1 Microbiology Department, School of Biomedical and Allied Health Sciences, University of Ghana, P.O. Box 4326, Accra, Ghana, West Africa; 2 Department of Microbiology, Korle-Bu Teaching Hospital, P.O. Box 77, Accra, Ghana, West Africa; 3 Department of Internal Medicine, La General Hospital, P.O.Box PMB La, Accra, Ghana, West Africa

**Keywords:** Ghana, Bloodstream, Infections, Infants, Adults, Antibiotic susceptibility

## Abstract

**Background:**

Bloodstream infections (BSI) are life-threatening emergencies. Identification of the common pathogens and their susceptibility patterns is necessary for timely empirical intervention.

**Methods:**

We conducted a 4-year retrospective analysis of blood cultures from all patients excluding neonates at the Korle-Bu Teaching hospital, Ghana, from January 2010 through December 2013. Laboratory report data were used to determine BSI, blood culture contamination, pathogen profile, and antimicrobial resistance patterns.

**Results:**

Overall, 3633 (23.16 %) out of 15,683 blood cultures were positive for various organisms. Pathogen-positive cultures accounted for 1451 (9.3 %, 95 % CI 8.5–9.8 %). Infants recorded the highest true blood culture positivity (20.9 %, n = 226/1083), followed by the elderly (13.3 %, n = 80/601), children (8.9 %, n = 708/8000) and adults (7.2 %, n = 437/6000) (*p* = 0.001 for Marascuilo’s post hoc). Overall occurrence of BSI declined with increasing age-group (*p* = 0.001) but the type of isolates did not vary with age except for *Citrobacter*, *Escherichia coli*, *Klebsiella*, *Salmonella*, and *Enterococcus* species. Gram negative bacteria predominated in our study (59.8 %, n = 867/1451), but the commonest bacterial isolate was *Staphylococcus aureus* (21.9 %, n = 318/1451)—and this trend run through the various age-groups. From 2010 to 2013, we observed a significant trend of yearly increase in the frequency of BSI caused by cephalosporin-resistant enterobacteria (Chi square for trend, p = 0.001). Meropenem maintained high susceptibility among all Gram-negative organisms ranging from 96 to 100 %. Among *Staphylococcus aureus*, susceptibility to cloxacillin was 76.6 %.

**Conclusion:**

Our study shows a significantly high blood culture positivity in infants as compared to children, adults and the elderly. There was a preponderance of *S. aureus* and Gram-negative bacteria across all age-groups. Meropenem was the most active antibiotic for Gram-negative bacteria. Cloxacillin remains a very useful anti-staphylococcal agent.

**Electronic supplementary material:**

The online version of this article (doi:10.1186/s12941-016-0163-z) contains supplementary material, which is available to authorized users.

## Background

Developing countries, especially in Africa, have a disproportionate burden of global bacterial bloodstream infections (BSI). This contributes significantly to morbidity, mortality, and increased health care costs [1–3]. The potential outcomes of bloodstream infections are such that treatment is often empirical, due to delays in performing and receiving blood culture results. In developing countries, this is further worsened by the unavailability of well-resourced microbiology facilities [4]. Empirical antibiotics used in such instances are usually based on international guidelines and not guided by local susceptibility data [5]. The rise in antibiotic resistance worldwide means that the likelihood of inadequate therapy in BSI is heightened, with increased chances of poor therapeutic outcomes [6, 7]. Knowledge of the local antibiotic resistance profile of bacteria increases the probability of selecting effective empirical therapy [2, 8]. In a recent study on the cost-effectiveness of bloodstream infection surveillance for the management of sepsis in low-resource settings, Penno et al. [5] showed that antibiotic prescribing, based on local antibiogram is able to save 534 additional lives per 100,000 patients. The finding suggests that it is crucial to monitor emerging trends in resistance at the local level to support clinical management. However, data on antimicrobial resistance, especially regarding trends in BSI, remain scarce in Ghanaian healthcare settings including Korle-Bu Teaching Hospital (KBTH).

Published data describing BSI in KBTH are limited to short study periods [9] and have mainly focused on children [10]. Using a large collection of blood culture data, we have examined trends in pediatric and adult BSI over 4 years at KBTH; determined blood culture yields and contamination rate; and analyzed the antibiotic susceptibility profiles of the associated organisms, with emphasis on multidrug resistant phenotypes of public health concern.

## Methods

### Study design

This was a hospital based retrospective analysis of blood culture reports from January 2010 to December 2013 at the Korle-Bu Teaching Hospital in Ghana, West Africa. We followed guidelines for the reporting of studies conducted using observational routinely-collected health data (RECORD) [11]. The KBTH is a 2000-bed tertiary teaching hospital with about 200 admissions per day [12]. The central outpatient department records about 29,757 patients per month [12]. The hospital covers all medical specialties and provides referral healthcare services to an estimated population of 24 million Ghanaians.

### Study settings and population

We collated blood culture results of patients older than 28 days, from the bacteriology unit of the Microbiology Laboratory in KBTH. Data on BSI of patients ≤28 days old will be published elsewhere. The bacteriology unit of KBTH processes over 40,000 clinical cultures annually. To obtain a good representation of the repertoire of BSI isolates occurring at a tertiary health facility, we have included all blood culture results of patients primarily receiving healthcare at the KBTH (n = 15,072) or secondarily referred to the laboratory from other health facilities for microbiological investigations (n = 611). We included data for the period January 2010 to December 2013. Data prior to this period were not properly archived and therefore difficult to access.

### Selection, review and extraction of data from laboratory reports

A data entry team comprising three bacteriologists and a physician who assisted with the medical reconciliation of data conducted a manual work-through of laboratory paper records to select blood cultures conducted between 2010 and 2013. Selected blood culture reports were reviewed and extracted into a computerized database (Statistical Package for Social Sciences, Version 20.0) as the primary data source. We had no independent means of validating laboratory results, thus reports were excluded from entry on the basis of incomplete data if results regarding the positivity or otherwise of blood cultures were inconclusive. The quality of abstracted data was assessed by an independent reviewer in a standardized manner by comparing paper records to the electronic entries. Disagreements between data were resolved by consensus between the reviewer and the data entry team. Extracted information included patient demographics, bacterial and fungal isolates, BSI categorization, and antibiogram of isolates. Reports for patients aged ≤28 days were not considered for analysis. True positive cultures were those with identified bacteria plus corresponding susceptibility results, or yeast. Organisms, including *Micrococcus species, Bacillus species,* and diphtheroids were classified as contaminants. For the majority of patients, only a single blood culture was submitted per infection episode. Where duplicate cultures were submitted, and the same organism was isolated within 14 days of the previous culture, then the latter isolate was excluded except when there was variation in antibiotic susceptibility pattern.

### Data source/methods of laboratory assessment for blood cultures

For patients with suspected sepsis, local guidelines recommended the inoculation of 1–3 mL (for paediatric patients; however, for older children, larger blood inoculums of 10 mL were encouraged) and 8–10 mL (for adults) directly into Bactec^®^ culture vials (Becton–Dickinson, USA). Routinely at the laboratory, cultures were processed with the BACTEC 9240 blood culture system (Becton–Dickinson, NJ, USA) according to manufacturer’s instructions. Where bacterial growth was detected in vials, Gram-stains were performed; and subcultures were typically made onto appropriate media based on Gram-stain results. Bacterial isolates were identified using routine biochemical methods. Bacteria speciation was done with the BBL^®^ Crystal identification system (Becton–Dickinson, NJ, USA). For positive fungal blood cultures, organisms were identified on the basis of morphology. As part of regular practice at the laboratory, consultant microbiologists evaluated all positive blood cultures to categorize isolates as contaminants or true pathogens. Susceptibility testing for bacterial pathogens were conducted using the Kirby-Bauer disc diffusion method with antibiotic discs; and these tests were interpreted according to guidelines by the Clinical and Laboratory Standards Institute (CLSI) [13].

#### Antibiotic resistance phenotypes

We assessed the occurrence of six epidemiologically important bacterial pathogens: vancomycin resistant *Enterococcus* species (VRE) [based on in vitro susceptibility to vancomycin disk (30 μg)], methicillin resistant *Staphylococcus aureus* (MRSA) [based on in vitro susceptibility to cefoxitin disk (30 μg)], penicillin resistant streptococci (PRS) [based on in vitro susceptibility to ampicillin disk (10 μg)], cephalosporin resistant enterobacteria (Ceph-R Ent) [based on in vitro susceptibility to cefotaxime disk (30 μg)], multi-drug resistant *Pseudomonas* species (MDR Ps.) and multi-drug resistant *Acinetobacter* species (MDR Act). A multidrug resistant (MDR) phenotype was defined, relative to the panel of antibiotics reported for each bacteria, as in vitro non-susceptibility to ≥1 agent in ≥3 antimicrobial classes: penicillins, cephalosporins, β-lactamase inhibitor combinations, carbapenems, tetracyclines, folate pathway inhibitors, glycopeptides, fluoroquinolones, chloramphenicol, aminoglycosides, and macrolides [14].

### Data analysis

Data analysis was performed with the Statistical Package for Social Sciences, Version 20.0. The distribution of BSI and resistance profiles of bacterial isolates were assessed using descriptive methods. Blood culture positivity and contamination levels were calculated by dividing positive blood cultures and contaminants respectively by the total number of blood cultures submitted. Susceptibility tests reports were classified as resistant or susceptible, and the data expressed as the proportion of susceptible isolates out of the total valid test results for the specific antibiotic and pathogen. Missing data were excluded from analysis. Study data were compared across four patient age categories: infants (>28 days–1 year), children (>1–15 years), adults (>15–65 years), and the elderly (>65 years). The Chi square analysis with Marascuilo’s post hoc tests for multiple comparisons was used to compare categorical data within patient groups. A Chi square test for linear trend was used to assess changes in categorical variables over the study period. Continuous data were compared using Students’ t test with analyses of variance (ANOVA) for multiple comparisons.

### Ethical considerations

During the data extraction process, we de-identified blood culture reports to ensure complete obscurity from laboratory archives. Arbitrary numbers were assigned to all reported isolates. Patients’ clinical data were not reviewed for analysis. Owing to the retrospective study design, and given the anonymized nature of our data, patients’ consent for use of the laboratory records could not be obtained. Ethical approval for isolates and laboratory data was not required as the study was regarded as part of routine surveillance measures for infection control. Study findings are freely available to the hospital for the formulation of guidelines for treatment of BSI.

## Results

**Fig. 1:**
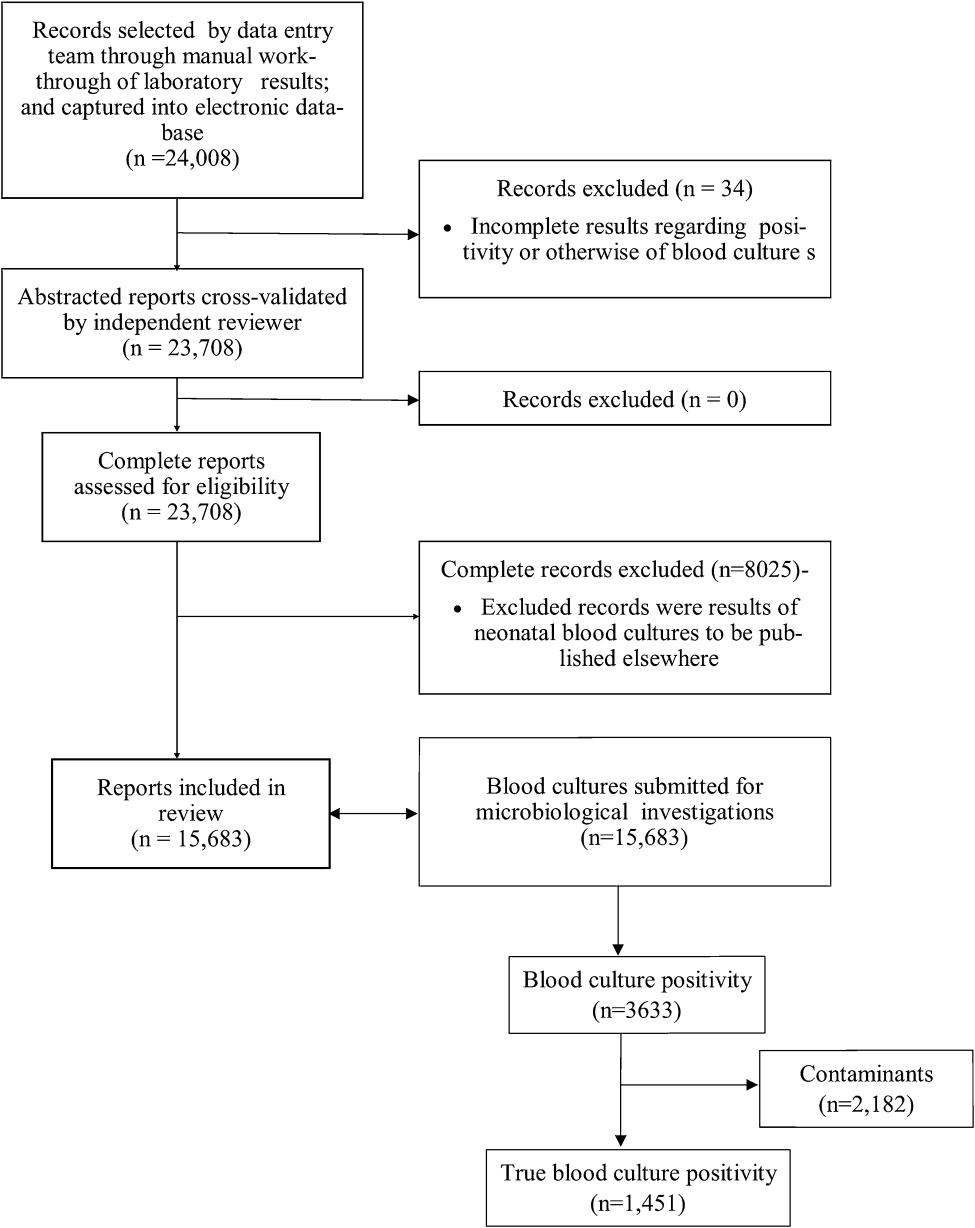
Flow diagram for data collation and results outcome. Overall, 24,042 laboratory records were reviewed, 15,683 of which belonged to patients >28 days and were eligible for inclusion. Of the 15,683 blood cultures submitted, 1451 were positive with various pathogens

Figure 1 shows the flow diagram for data collation and outcome. Overall, a total of 15,683 blood cultures were performed for infants (n = 1082), children (n = 8003), adults (n = 5987), and the elderly (n = 601). Males comprised 53 % (n = 8312) of patients. The median age of the study population was 31.6 years (range 29 days–69 years). About 3633 (23.16 %) blood cultures were positive: 1451 (9.3 %, 95 % CI 8.5–9.8) were considered true pathogens and 2182 (13.9 %) considered contaminants. The rate of BSI was 92.5 per 1000 blood cultures submitted to the laboratory, with no significant variation across the years under review (2010, 82.4 per 1000 blood cultures; 2011, 94.2 per 1000 blood cultures; 2012, 87.2 per 1000 blood cultures; 2013, 90.3 per 1000 blood cultures: *p* = 0.0841 for Marascuilo’s post hoc). The median age of patients with BSI was 13.5 years (range 29–81 years). Chi square for linear trend analysis showed that the overall occurrence of BSI declined with increasing age-group (*p* = 0.001), but most of the isolates did not vary with age (Table 1). The highest true blood culture positivity was observed among infants (20.9 %, n = 226/1083), followed by the elderly (13.3 %, n = 80/601), children (8.9 %, n = 708/8000) and adults (7.2 %, n = 437/6000) (*p* = 0.001 for Marascuilo’s post hoc). The proportion of blood cultures positive for various pathogens remained similar (*p* = 0.412 for Chi square test) between patients receiving care at KBTH (9.3 %, n = 1407/15,072) and those referred to the laboratory from other health facilities (7.6 %, n = 44/611). In the proceeding analysis, we have disregarded comparing results between the two sources of blood cultures because there were few true positive cultures from the latter.

**Table 1:** Distribution of BSI isolates across age groups

Organism (n, %)	Infants (A) (29 days–1 year)	Children (B) (1–15 years)	Adults (C) (15–65 years)	Elderly (D) (>65 years)	Post hoc comparison	trend
GNB (n = 867, 59.8 %)	123 (54.4)	407 (57.5)	265 (65.4)	52 (65.0)	A = B = C = D	0.002
Enterobacteria (n = 640, 44.1 %)	81 (35.8)	312 (44.1)	203 (46.4)	44 (55.0)	A < D = B = C	0.001
*Citrobacter* sp. (n = 148, 10.2 %)	14 (6.2)	58 (8.1)	65 (13.0)	11 (13.7)	A = B = C = D	0.001
*E. coli* (n = 118, 8.1 %)	8 (3.5)	27 (3.8)	60 (13.7)	23 (28.7)	A = B < C = D*	0.001
*Enterobacter* sp. (n = 105, 7.2 %)	14 (6.2)	53 (7.5)	32 (7.3)	6 (7.5)	A = B = C = D	0.731
*Klebsiella* sp. (n = 63, 4.3 %)	16 (7.1)	30 (4.2)	14 (3.2)	3 (3.8)	A = B = C = D	0.037
*Proteus* sp. (n = 9, 0.6 %)	2 (0.8)	3 (0.4)	4 (0.9)	0	A = B = C = D	0.668
*Providencia* sp. (n = 4, 0.4 %)	1 (0.4)	1 (0.1)	2 (0.5)	0	A = B = C = D	0.738
*P. agglomerans* (n = 2, 0.1 %)	0	1 (0.1)	1 (0.2)	0	A = B = C = D	0.992
*Salmonella* sp. (169, 11.6 %)	23 (9.7)	127 (17.9)	18 (4.3)	1 (1.3)	B > C = A > D*	0.001
*Serratia* sp. (n = 1, 0.1 %)	0	0	1 (0.2)	0	A = B = C = D	0.755
Other coliform (n = −21, 1.4 %)	3 (1.3)	12 (1.7)	6 (1.4)	0	A = C = D < B	0.429
Non-fermentative bacilli (n = 223, 15.3 %)	42 (18.5)	92 (12.9)	81 (18.5)	8 (10.0)	A = B = C = D	0.818
*Acinetobacter* sp. (n = 120, 8.3 %)	24 (10.6)	56 (7.9)	36 (8.2)	4 (5.0)	A = B = C = D	0.173
*Pseudomonas* sp. (n = 101, 7.0 %)	18 (7.9)	35 (4.9)	44 (10.0)	4 (5.0)	A = B < C = D*	0.404
*S. paucimobilis* (n = 1, 0.1 %)	0	1 (0.1)	0	0	A = B = C = D	0.334
*S. maltophilia* (n = 1, 0.1 %)	0	0	1 (0.2)	0	A = B = C = D	0.759
Other gram negatives (n = 4, 0.2 %)	0	3 (0.4)	1 (0.2)	0	A = B = C = D	0.738
*Chromobacter* sp. (n = 1, 0.1 %)	0	0	1 (0.2)	0	A = B = C = D	0.759
*N. meningitides* (n = 2, 0.2 %)	0	3 (0.4)	0	0	A = B = C = D	0.350
GPB (n = 563, 36.5 %)	102 (45.1)	289 (40.7)	146 (25.9)	26 (32.5)	C < A = B = D	0.0001
*Enterococcus* sp. (n = 43, 0.3 %)	8	24 (3.4)	10 (22.8)	1 (1.3)	A = B = C = D	0.139
*S. aureus* (n = 318, 21.9 %)	60 (26.5)	148 (20.9)	99 (22.6)	11 (13.8)	A = B = C = D	0.092
*S. epidermidis* (n = 67, 4.6 %)	11 (4.8)	42 (5.9)	12 (2.7)	2 (2.6)	A = B = C = D	0.047
*S. agalactiae* (n = 1, 0.1 %)	1 (0.4)	0	0	1 (1.3)	A = B = C = D	0.984
*S. pneumoniae* (n = 35, 2.4 %)	6 (2.6)	23 (3.2)	3 (0.6)	3 (3.8)	A = B < C = D*	0.159
*S. pyogenes* (n = 3, 0.2 %)	0	1 (0.1)	1 (0.2)	1 (1.3)	A = B = C = D	0.217
*S. viridans* (n = 48, 3.3 %)	10 (4.4)	23 (3.2)	9 (8.9)	6 (7.5)	A = B = C = D	0.739
Other *Streptococcus* sp. (n = 48, 3.3 %)	7 (3.1)	28 (4.0)	12 (2.7)	1 (1.3)	A = B = C = D	0.243
Fungi (n = 21, 1.3 %)	2 (0.8)	12 (1.7)	5 (1.1)	2 (2.6)	A = B = C = D	0.596
*Aspergillus* sp. (n = 3, 0.2 %)	0	2 (0.2)	0	1 (1.3)	A = B = C = D	0.588
*Candida* sp. (n = 17, 1.2 %)	2 (0.8)	10 (1.4)	4 (0.9)	1 (1.3)	A = B = C = D	0.816
*Cryptococcus* sp. (n = 1, 0.1 %)	0 (0)	0	1 (0.2)	0	A = B = C = D	0.759
True blood culture positivity (n = 1451, 9.3 %)	226 (20.9 %)	708 (8.9 %)	437 (7.3 %)	80 (13.3 %)	A > D > B > C*	<0.001

Sp., species; GNB, gram negative bacteria; GPB, gram positive bacteria;* E. coli*, *Escherichia coli*; *P. agglomerans*, *Pantoea agglomerans*;* S. paucimobilis*, *Sphingomonas paucimobilis*;* S. maltophilia*, *Stenotrophomonas maltophilia*;* N. meningitis*, *Neisseria meningitis*; *S. aureus*, *Staphylococcus aureus*;* S. epidermidis*, *Staphylococcus epidermidis*;* S. agalactiae*, *Streptococcus agalactiae*;* S. pneumoniae*, *Streptococcus pneumoniae*;* S. pyogenes*, *Streptococcus pyogenes*;* S. viridans*, *Streptococcus viridans*;  *trend*, Chi square for linear trend; Post hoc, comparison compares the proportion of bloodstream infections among age-groups

* Comparisons with significant (p < 0.05) differences

### Distribution of isolates

Gram-negative bacteria accounted for 59.8 % (n = 867/1451) of BSI pathogens, whilst Gram-positives and fungi accounted for 38.8 % (n = 563/1451) and 1.4 % (n = 21/1451) respectively (*p* = 0.001 for Marascuilo’s post hoc)—and the trend was similar within each age-group. *Staphylococcus aureus* was the most isolated organism (21.9 %, n = 318/1451). The majority of Gram-negative bacteria belonged to the family *Enterobacteriaceae* (73.9 %, n = 640/867), with *Salmonella* (11.1 %) and *Citrobacter* (10.2 %) species being the second and third most isolated pathogens (Table 1).

**Table 2:** Percentage susceptibility of gram negative pathogens

Organism	Amp	Am/cl	Gen	Amk	Cip	Lev	Cef	Ctx	Mem	Cot	Tet	Chl
*Citrobacter* sp.	6/120 (5)	8/33 (24.2)	44/133 (33.1)	100/118 (84.7)	46/64 (71.9)	19/27 (70.4)	34/136 (25)	46/142 (32.4)	148/148	19/113 (16.8)	15/103 (14.6)	11/96 (11.5)
*Serratia* sp.	0/1		1/1	1/1	1/1		1/1	1/1	1/1	0/1	1/1	1/1
*E. coli*	2/86 (2.3)	5/40 (12.5)	40/86 (46.5)	67/87 (77)	25/66 (37.9)	9/31 (29)	31/104 (29.8)	49/112 (43.8)	118/118 (100)	6/79 (7.6)	6/69 (8.7)	8/71 (11.3)
*Enterobacter* sp.	2/91 (2.2)	2/29 (6.9)	27/86 (31.4)	68/89 (76.4)	37/57 (64.9)	16/23 (69.6)	19/98 (19.4)	22/100 (22)	104/105 (99)	14/81 (17.3)	7/71 (9.9)	7/64 (10.9)
*Klebsiella* sp.	0/56 (0)	3/18 (16.7)	11/52 (21.2)	43/53 (81.1)	20/33 (60.1)	12/19 (63.2)	9/59 (15.3)	12/60 (20)	62/63 (98.4)	7/53 (13.2)	3/45 (6.7)	6/44 (13.6)
*Proteus* sp.	0/6 (0)	2/4 (50)	5/7 (71.4)	5/6 (83.3)	4/5 (80)	1/2 (50)	5/9 (5.6)	6/9 (66.7)	9/9	1/6 (16.7)	0/3 (0)	1/5 (20)
*Providencia* sp.	0/3		3/4 (75)	4/4	1/1		1/4 (25)	2/4 (50)	4/4	0/4	1/3 (33.3)	0/5
*P. agglomerans*	0/2		1/2 (50)	2/2			0/2	0/2	2/2	0/2	0/2	
*Salmonella* sp.	48/133 (36.1)	35/46 (76.1)	125/133 (94)	131/133 (98.5)	96/96 (100)	40/41 (97.6)	130/156 (83.3)	154/162 (95.1)	169/169	59/122 (48.3)	35/106 (33)	29/111 (26.1)
*Serratia* sp.	0/1(0)		1/1	1/1	1/1		1/1	1/1	1/1	0/1	1/1	1/1
*Acinetobacter* sp.	4/56 (7.1)	6/16 (37.5)	25/55 (45.5)	43/57 (75.4)	18/25 (72)	10/17 (58.8)	17/66 (25.8)	16/70 (22.9)	116/120 (96.7)	12/51 (23.5)	3/50 (6)	6/50 (12)
*Pseudomonas* sp.			45/67 (67.2)	53/61 (86.9)	41/51 (80.4)	39/49 (79.6)			97/101 (96)			
*S. maltophilia*			0/1	0/1		1/1			1/1			
*S. paucimobilis*	1/1		1/1	1/1			1/1	1/1	1/1	0/1	0/1	
*Chromobacter* sp.		0/1		1/1		1/1	0/1	1/1	1/1			
*N. meningitidis*	1/1	1/1			2/3 (66.7)		2/3 (66.7)	3/3		0/1		0/1

*Amp* ampicillin,* Am/cl* amoxicillin clavulanic acid,* Gen* gentamicin,* amk* amikacin,* cip* ciprofloxacin,* Lev* levofloxacin,* Cef* cefuroxime,* ceft* cefotaxime,* Mem* meropenem,* Cot* cotrimoxazole,* Tet* tetracycline,* Chl* chloramphenicol,* sp* species, *E. coli* *Escherichia coli*, *P. agglomerans* *Pantoea agglomerans*, *S. paucimobilis* *Sphingomonas paucimobilis*,*S. maltophilia* *Stenotrophomonas maltophilia*,*N. meningitis* *Neisseria meningitis*

**Table 3:** Percentage susceptibility of gram positive pathogens

Organisms	Pen	Amp	Amc/cl	Gen	Cef	Ox	Vanc	Ery	Tet	Ctx	Cot	Chl
*Staphylococcus epidermidis*						3/6 (50)	4/4 (100)					
*Staphylococcus aureus*	20/293 (6.8)	26/260 (10)	42/60 (70)	170/250 (68)	141/291 (48.5)	226/295 (76.6)	144/144 (100)	111/182 (70)			59/242 (24.4)	89/233 (38.2)
*Enterococcus* sp.	1/38 (2.6)	3/39 (7.7)					15/17 (88.2)		14/33 (42.4)			
*Streptococcus pyogenes*	3/3 (100)	2/2 (100)	2/2 (100)		3/3 (100)		2/2 (100)	2/2 (100)		1/1 (100)		
*Streptococcus viridans*	4/45 (8.9)	7/42 (16.6)	5/13 (34.5)		16/40 (.4)		19/20 (95)	5/20 (25)		28/40 (70)		4/25 (16)
*Streptococcus agalactiae*	0/1 (0)	0/1 (0)			0/1 (0)		0/1 (0)	0/1 (0)				
*Streptococcus pneumoniae*	10/31 (32.5)	14/30 (46.7)	10/10 (100)		27/39 (69.2)		12/12 (100)	16/19 (84.2)		32/32 (100)		4/19 (21.1)
*Streptococcus* sp.	3/41 (7.3)	6/42 (14.3)	6/9 (66.7)		20/43 (46.5)		24/27 (88.9)	15/28 (53.8)		12/23 (52.2)		12/35 (34.3)

Data not available for blank cells

*Pen* penicillin,* Amp* ampicillin,* Am/cl* amoxicillin clavulanic acid,* Gen* gentamicin,* Cef* cefuroxime,* Cot* cotrimoxazole,* Tet* tetracycline,* Chl* chloramphenicol,* Ctx* cefotaxime,* Chl* chloramphenicol

### Antibiotic susceptibility

Our results show high levels of ampicillin resistance among Gram-negative (Table 2) and Gram-positive (Table 3) organisms. Susceptibility among the enterobacteria ranged from 0 % among *Proteus* and *Klebsiella* species to 36.1 % in *Salmonella* species. There was 100 % susceptibility among *Neisseria meningitides* isolates. Among Gram-positive organisms, susceptibility ranged from 7.7 % in *Enterococcus* species to 100 % in *Streptococcus pyogenes*. Among the aminoglycosides, Gram-negative organisms maintained high susceptibility to amikacin than to gentamicin. For *Pseudomonas* species, susceptibility of 86.9 and 67.2 % were respectively recorded for amikacin and gentamicin, whilst for *Citrobacter* species, susceptibility of 84.7 and 33.1 % were noted. In this study, moderate to high level susceptibility of Gram-negative organisms to quinolones was recorded, exemplified by 64.9 and 69.6 % susceptibility of *Citrobacter* species, as well as 100 and 97.6 % susceptibility of *Salmonella* species, to ciprofloxacin and levofloxacin respectively. Most enterobacteria showed low to moderate (20–43 %) susceptibility to cefotaxime. High percentage susceptibility were however recorded for *Salmonella* (95.5 %). Meropenem retained high susceptibility among all Gram-negative organisms, ranging from 96 % among *Pseudomonas* species to 100 % among *Citrobacter* species. Among *Staphylococcus aureus*, susceptibility to cloxacillin over the period was 76.6 %, cefuroxime 48.5 %, erythromycin 70 %, co-trimoxazole 24 %, gentamicin 68 % and vancomycin 100 %. Isolates of *Streptococcus pneumoniae* were moderately susceptible to penicillin (32.5 %) and ampicillin (46.7), but highly susceptible to erythromycin (84.2 %) and vancomycin (100 %).

### Trends in antibiotic susceptibility patterns

**Fig. 2:**
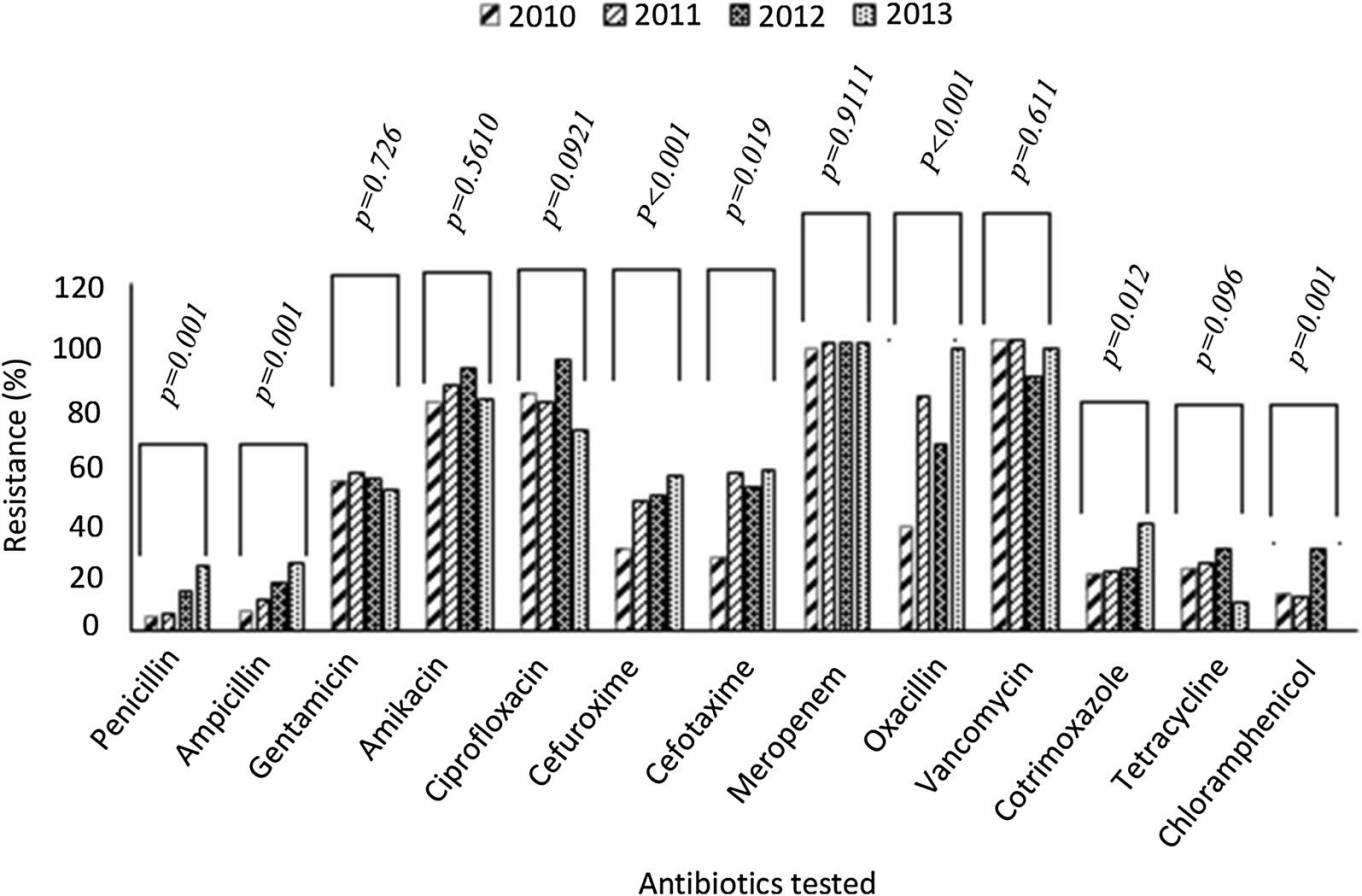
Trends in antibiotic susceptibility patterns over 4 years (2010 through to 2013). *p* values calculated for Mantel–Haenszel Chi square for linear trends

**Fig. 3:**
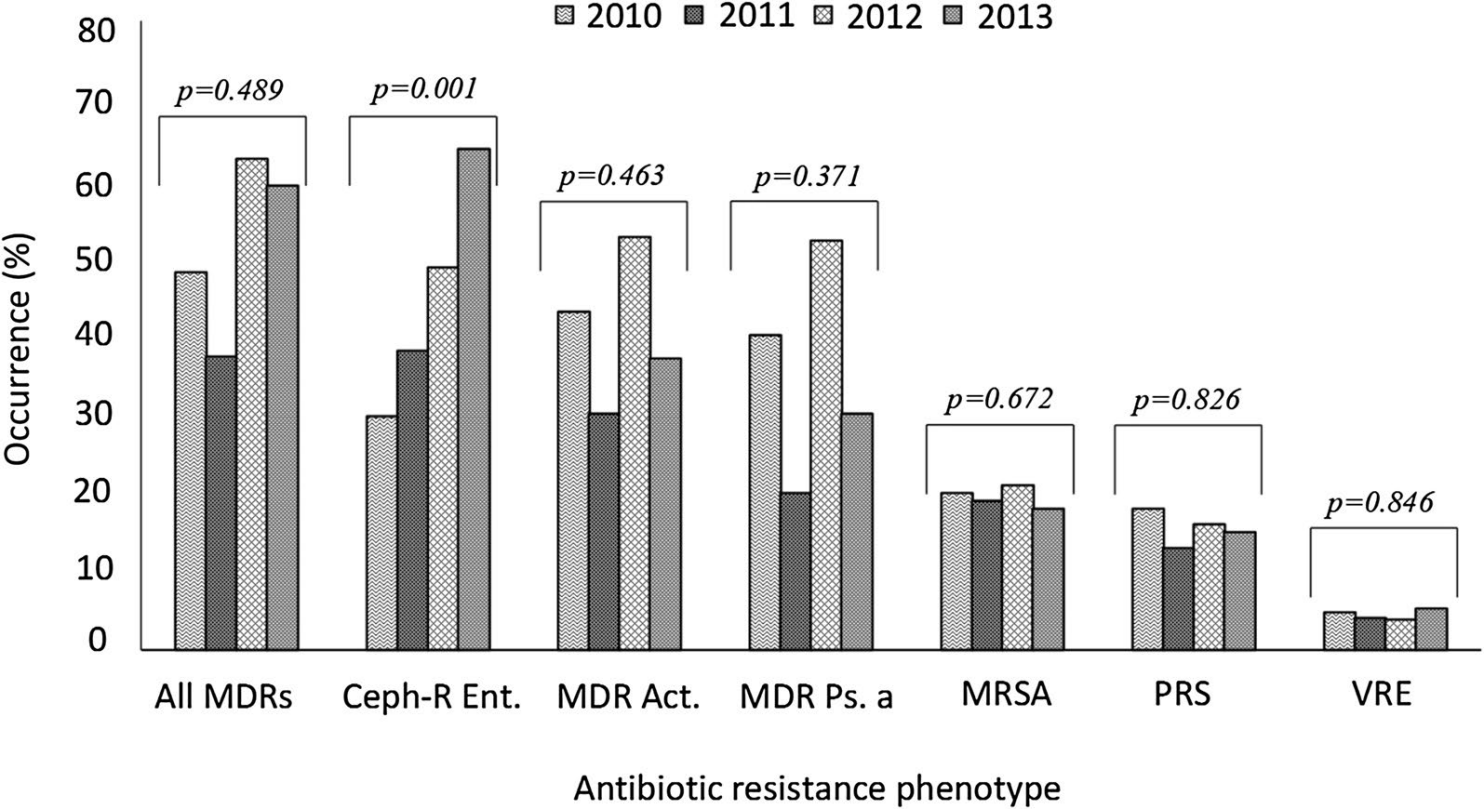
Antimicrobial resistance phenotypes over 4 years (2010 through to 2013). *p* values calculated for Mantel–Haenszel Chi square for linear trends. *MDRs* multidrug resistant bacteria, *VRE* vancomycin resistant Enterococci, *MRSA* methicillin resistant *Staphylococcus aureus*, *PRS* penicillin resistant Streptococci, *Cef-R Ent* cephalosporin resistant Enterobacteriaceae, *MDR Ps* multidrug resistant *Pseudomonas aeruginosa*, *MDR Act* multidrug resistant Acinetobacter species

In Fig. 2, we assessed antibiotic susceptibility patterns over 4 years, looking out for significant trends. There was a significant rise in susceptibility to penicillin (*p* = 0.001), ampicillin (*p* = 0.000), cefuroxime (0.0000), cloxacillin (*p* = 0.0000), cotrimoxazole (*p* = 0.012), cefotaxime (*p* = 0.0197) and chloramphenicol (*p* = 0.001). Figure 3 shows the occurrence of some epidemiologically important antibiotic resistance phenotypes: VRE, MRSA, PRS, Cef-R Ent, MDR Ps, MDR Act, and all MDRs. From 2010 to 2013, we observed a significant trend towards increasing frequency of BSI caused by Cef-R Ent (Chi square for trend, *p* = 0.001). No other resistant phenotype varied with years.

## Discussion

Bloodstream infections accounted for 9.3 % of blood cultures and varied significantly within age-groups, where the highest prevalence was recorded among patients at the extremes of ages: the elderly (13.3 %) and infants (20.9 %). The reasons for this distribution is unclear, as risk factors for developing BSI were not assessed in this study. Nevertheless, infants were significantly more likely to have BSI compared with patients aged >3 years. There may be several reasons for this association. Infants are likely to have frequent exposure to healthcare environments or participate in activities that predispose them to microbial contamination. They also have poor skin integrity and an immature immune system [15–19].

In keeping with previous studies from Ghana [20, 21], Gram-negative bacteria predominated in our study, but the majority of bacterial findings were *S. aureus*, which also predominated in other studies from Guinea-Bissau [22], Nigeria [16] and Malawi [23]. We believe that the aetiology of BSI is changing, so that BSI are due largely to Gram-negative bacteria, but with a significant contribution from Gram-positive isolates, mostly *Staphylococcus* species. Other studies in Africa have documented this phenomenon, attributing the predominance of Gram-positive bacteria (mostly *S. pneumoniae and S. aureus*) to community-acquired infections [24–26] and Gram-negative bacteria (predominantly *E. coli and K. pneumoniae*) to healthcare related infections [27]. We are unable to determine which BSI were healthcare related or community-acquired because of insufficient data on inpatient and outpatient status. The presence of *Streptococcus pneumoniae* as a cause of BSI was uncommon in our study. This could partially be attributable to pneumococcal vaccine introduced in January 2012. Immunization of infants with the vaccine has led not only to major declines in childhood invasive pneumococcal sepsis but also to a reduction in infections among adults due to herd immunity [27]. It is the experience in KBTH that *Salmonella* species constitute a prominent pathogen in BSI, with a preponderance of non-typhoidal salmonellae over typhoidal salmonellae. Children under 5 years bear the brunt of the disease [28]. These observations are generally consistent with findings from other African studies, particularly in malaria-endemic regions, where *Salmonella* species have predominated in BSI [29, 30].

Favoured by of their low toxicity, broad spectrum and comparatively high effectiveness, the cephalosporin antibiotics are widely used in Ghana. A worrying observation is the increasing isolation of cephalosporin resistant *Enterobacteriaceae* over the 4-year review period. The enterobacteria constituted over 40 % of the bloodstream pathogens. Given that the variety of class C cephalosporinases (AmpCs) and extended spectrum β-lactamases (ESBLs) remain the principal mechanisms for cephalosporin resistance worldwide [31–33], the high levels of cephalosporin resistance observed in our study may be compatible with results from other studies in Ghana, demonstrating high levels of ESBLs (>45 %) among the enterobacteria [31, 34].

Based on the low frequency of fungi and the dominance of both GPB and GNB, it may be necessary for clinicians to consider suitable empiric regimens that provide adequate cover for both bacterial groups. Cefotaxime, gentamicin, ciprofloxacin and amoxicillin plus clavulanic acid, in various combinations, have been the predominant empiric therapies for bloodstream infections in Ghana [35]. We have demonstrated here and in previous studies that the general antibiogram of the GNB and GPB reveal an overall high resistance to these routinely used drugs [31, 32]. Our observations are in consonance with other studies in Ghana and at Korle-Bu teaching Hospital, which found high resistance to ampicillin, gentamicin and cefotaxime among bacteria isolates of various infections [31, 32, 36]. Carbapenem was the most active antibiotic against Gram-negative infections. Meanwhile, cloxacillin remained a very useful anti-staphylococcal agent due the relatively low prevalence of MRSA.

This study has several limitations. Being retrospective, we were unable to ascertain the standardization of techniques for blood cultures and laboratory procedures for individual patients. The study is also unable to evaluate the accuracy of bacteria isolates classified as contaminants in laboratory records. As the study lacks comprehensive clinical data or information on recent antibiotic use and/or hospitalization, our data on the occurrence of BSI should be interpreted with caution. Finally, our study was performed in a single center, and cannot be generalized to the entire patient population in Ghana. Our study nonetheless provides relevant data which may be used to guide empirical therapy, change antibiotic prescription policy in our institution or serve as a baseline for future research. We recommend future studies to ascertain the changing eligibility of our findings over time beyond the review years reported in this study.

## Conclusion

Our study shows that blood culture positivity was significantly higher in infants compared to children, adults and the elderly; with a preponderance of *S. aureus* and Gram-negative bacteria in all age-groups. The findings presented here suggest the need to review empiric antibiotic options in these patient populations, given the relatively low susceptibility of the bacterial isolates to many routinely used antibiotics.

## Additional file

10.1186/s12941-016-0163-z The RECORD statement—checklist of items, extended from the STROBE statement that should be reported in observational studies using routinely collected health data.

## Abbreviations

µg: microgram
ANOVA: analysis of variance
BSI: blood stream infections
CI: confidence interval
CLSI: Clinical and Laboratory Standards Institute
GNB: gram-negative bacteria
GPB: gram-positive bacteria
KBTH: Korle-Bu Teaching Hospital
MDR: multidrug resistance
SPSS: statistical package for social sciences
NJ: New Jersey
USA: United States of America
VRE: vancomycin resistant *Enterococcus* species
MRSA: methicillin resistant *Staphylococcus aureus*
PRS: penicillin resistant streptococci
Ceph-R Ent: cephalosporin resistant enterobacteria
MDR: multidrug resistant
MDR Ps: multi-drug resistant *Pseudomonas* species
MDR Act: multi-drug resistant *Acinetobacter* species
